# Positive effect of sublingual nitroglycerin on performance of non-contrast enhanced magnetic resonance coronary angiography

**DOI:** 10.1186/1532-429X-17-S1-P155

**Published:** 2015-02-03

**Authors:** Tobias Heer, Stephanie Reiter, Katrin Hauck, Berthold Höfling, Guenter Pilz

**Affiliations:** Cardiology, Clinic Agatharied, Hausham, Germany

## Background

Cardiac magnetic resonance imaging (CMR) is increasingly proposed for non-invasive detection of relevant coronary artery disease (CAD), currently using an integrated assessment of myocardial perfusion, viability and function. Magnetic resonance coronary artery imaging (MRCA) is still experimental. Currently, it is not proven if sublingual nitroglycerin (slNitro) should be given to enhance performance of MRCA.

## Methods

15 volunteers, 9 (60%) of them male, mean age 37.2 years (±11) without known CAD underwent MRCA, without and with slNitro. slNitro dosage was adjusted according to blood pressure (systolic blood pressure (BP) >180 mmHg =>1.6 mg glyceroltrinitrat, BP 140-179 mmHg =>1.2 mg, BP 100-139 mmHg =>0.8 mg, BP 90-100mmHg =>0.4mg). All volunteers were examined in supine position using a GE Signa HDxt 1.5 Tesla scanner, equipped with EchoSpeed gradients, and a dedicated 8-element phased array cardiac coil (GE Healthcare, Milwaukee, Wisconsin). For imaging of the coronary arteries a commercially available whole heart 3D navigator gated multislab steady state free precession sequence (3D HEART, based on 3D FatSat FIESTA) without the administration of contrast medium was employed. This sequence is designed for free breathing cardiac MR angiography, using a navigator echo pulse that detects motion of the diaphragm. We used an abdominal belt in all vol to reduce motion of the diaphragm. Post processing was perfomed with cvi^42^ 5.0 (Circle, Calgary, Canada). Vessel diameter was measured before and after slNitro. Image quality was graded visually before and after slNitro on a 4-point scale: 1 = non-assessable with severe image artifacts, poor vessel contrast; 2 = assessable with moderate image artifacts, fair vessel contrast; 3 = assessable with minor artefacts, good vessel contrast; and 4 = assessable with no apparent artifacts, excellent vessel contrast.

## Results

MRCA was successful in all volunteers and all coronary segments were assessable (IQ ≥2). Mean visible length of the right coronary artery (RCA) was 117 mm before, and 135 mm after slNitro (p=0.008), for the left anterior descending artery (LAD) it was 74 vs. 88 mm (p=0.001), and for the left circumflex artery (LCX) it was 52 vs. 65 mm (p=0.01). We compared vessel diameter and image quality (IQ) before and after slNitro in proximal, medial and distal segments (see table). Coronary artery diameter increased in all segments. IQ improved in medial (RCA and LCX) and distal segments, but not in proximal segments.

**Table 1:** Effect of nitroglycerin on performance of MRCA

Coronary vessel	Diameter before slNitro (mm, mean)	Diameter after slNitro (mm, mean)	P value	Image quality before nitro (mean)	Image quality after nitro (mean)	P value
RCA prox	3.8	4.2	0.001	2.7	2.9	0.19
RCA med	3.2	3.8	0.008	2.1	2.5	0.048
RCA dist	3.1	3.5	0.002	2.1	2.5	0.02
Left main	4.0	4.6	<0.001	3.4	3.5	0.3
LAD prox	3.4	4.0	<0.001	3.1	3.3	0.1
LAD med	3.1	3.6	<0.001	2.7	3.1	0.06
LAD dist	2.6	3.2	<0.001	2.1	2.7	0.006
LCX prox	3.2	3.6	0.01	2.4	2.7	0.1
LCX med	2.8	3.3	<0.001	2.4	2.9	0.004
LCX dist	2.5	3.9	0.02	2.0	2.5	0.004

## Conclusions

Non-contrast enhanced MRCA with abdominal belt was feasible in all volunteers and all coronary segments were assessable. After slNitro the visible length of all coronary arteries and the diameter of all coronary segments significantly improved. IQ increased in medial (RCA and LCX) and distal segments. Hence, our data provide preliminary evidence that MRCA should be performed after slNitro.

## Funding

None.

**Figure 1:**
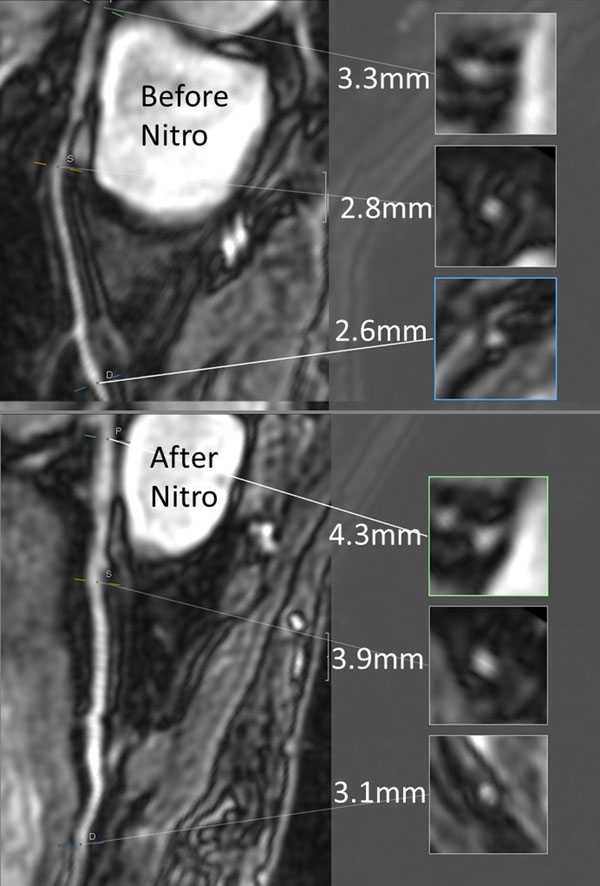
Effect of slNitro on vessel diameter - LAD before and after slNitro

